# Plasma Brain-Derived Neurotrophic Factor and Reverse Dipping Pattern of Nocturnal Blood Pressure in Patients with Cardiovascular Risk Factors

**DOI:** 10.1371/journal.pone.0105977

**Published:** 2014-08-25

**Authors:** Manabu Kadoya, Hidenori Koyama, Akinori Kanzaki, Masafumi Kurajoh, Miki Hatayama, Jun Shiraishi, Hirokazu Okazaki, Takuhito Shoji, Yuji Moriwaki, Tetsuya Yamamoto, Masaaki Inaba, Mitsuyoshi Namba

**Affiliations:** 1 Department of Internal Medicine, Division of Diabetes, Endocrinology and Metabolism, Hyogo College of Medicine, Nishinomiya, Hyogo, Japan; 2 Department of Endocrinology, Metabolism and Molecular Medicine, Osaka City University Graduate School of Medicine, Osaka, Japan; Rutgers University, United States of America

## Abstract

**Context:**

Basic studies have shown that brain-derived neurotrophic factor (BDNF) has critical roles in the survival, growth, maintenance, and death of central and peripheral neurons, while it is also involved in regulation of the autonomic nervous system. Furthermore, recent clinical studies have suggested potential role of plasma BDNF in the circulatory system.

**Objective:**

We investigated the mutual relationships among plasma BDNF, patterns of nocturnal blood pressure changes (dippers, non-dippers, extra-dippers, and reverse-dippers), and cardiac autonomic function as determined by heart rate variability (HRV).

**Design:**

This was a cross-sectional study of patients registered in the Hyogo Sleep Cardio-Autonomic Atherosclerosis (HSCAA) Study from October 2010 to November 2012.

**Patients:**

Two-hundred fifty patients with 1 or more cardiovascular risk factor(s) (obesity, smoking, presence of cardiovascular event history, hypertension, dyslipidemia, diabetes mellitus, chronic kidney disease) were enrolled.

**Results:**

Plasma BDNF levels (natural logarithm transformed) were significantly (p = 0.001) lower in reverse-dipper patients (7.18±0.69 pg/ml, mean ± SD, n = 36) as compared to dippers (7.86±0.86 pg/ml, n = 100). Multiple logistic regression analysis showed that BDNF (odds ratios: 0.417, 95% confidence interval: 0.228–0.762, P = 0.004) was the sole factor significantly and independently associated with the reverse-dippers as compared with dippers. Furthermore, plasma BDNF level was significantly and positively correlated with the time-domain (SDNN, SDANN5, CVRR) and frequency-domain (LF) of HRV parameters. Finally, multiple logistic regression analyses showed that the relationship between plasma BDNF and the reverse-dippers was weakened, yet remained significant or borderline significant even after adjusting for HRV parameters.

**Conclusions:**

Low plasma BDNF was independently associated with patients showing a reverse-dipper pattern of nocturnal blood pressure, in which an imbalance of cardiac autonomic function may be partly involved.

## Introduction

Brain-derived neurotrophic factor (BDNF), originally discovered in the brain and reported to be a member of the neurotrophin family [1], exerts its effects by activating the tropomyosin-related kinase receptor B (TrkB) [2]. It has been shown to be expressed in the central and peripheral nervous systems, and able to cross the blood-brain barrier in both directions [3]. BDNF has been reported to have critical roles in the survival, growth, maintenance, and death of central and peripheral neurons, and is also present in systemic circulation [4].

Considerable evidence has been presented showing that BDNF has essential roles in energy homeostasis [5]. Heterozygous BDNF deficiency in mice results in hyperphagia and obesity [6], while peripheral injection of the factor is anorexigenic [7]. Moreover, severe hyperphagia and obesity develop in individuals with BDNF haploinsufficiency, or a missense mutation of the TrkB gene in human [8], [9]. Besides roles in energy homeostasis, BDNF appears to be essential for regulation of the cardiovascular system as it is involved in development and survival of the arterial baroreceptor system [10], [11], and injection into the rostral ventrolateral medulla increases arterial blood pressure [12]. Furthermore, this factor was recently reported to have important protective roles against atherosclerotic plaque instability [13] and cardiac dysfunction [14]. Plasma BDNF levels are known to increase as a result of neural signals after myocardial infarction and its up-regulation appears to be critical to protect the myocardium against ischemic injury [14]. Thus, BDNF has attracted considerable attention as a key factor linking neuronal and cardiovascular regulation.

In spite of accumulated findings from animal studies, evidence for the significance of plasma BDNF level in the human cardiovascular system is quite limited. BDNF expression was found to be significantly increased in atherosclerotic coronary arteries, as compared to non-atherosclerotic coronary arteries from control subjects [13]. One study has shown that plasma BDNF levels are decreased in patients with acute coronary syndromes [15]. Recently, plasma BDNF levels were measured in a cohort of healthy subjects enrolled in the Baltimore Longitudinal Study of Aging (BLSA) and found to be correlated with blood pressures [16]. These basic and clinical findings of BDNF led us to examine plasma BDNF in relation to diurnal and nocturnal changes in blood pressure (BP) [17], as well as cardiac autonomic function determined by heart rate variability (HRV).

In healthy subjects, BP falls by 10% to 20% during sleep as compared to awake. However, there are several abnormal nocturnal BP fall patterns, with affected individuals classified as extreme-dippers if the fall is ≥20%, non-dippers if the fall is ≥0% but <10%, and reverse-dippers if the fall is <0% [18]. Previous studies have shown that non- and reverse-dippers with nocturnal hypertension are associated with elevated risk for cardiovascular diseases including stroke and mortality [18]. As a potential mechanism of nocturnal hypertension, dysfunction of the autonomic nervous system may mediate the influences of stress, sleep disorders, and deterioration of the endocrine system [19]–[21]. In the present study, the mutual relationships among plasma BDNF, HRV, as a measure of cardiac autonomic function, and nocturnal BP changes (extreme-dipper, dipper, non-dipper, reverse-dipper) were examined in 250 patients with cardiovascular risk factors.

## Methods

### 1. Study design and participants

This cross-sectional study was conducted from October 2010 to November 2012 and included 250 registered patients who were part of the Hyogo Sleep Cardio-Autonomic Atherosclerosis (HSCAA) Study, which was designed to examine the impacts of sleep, autonomic imbalance, and subclinical atherosclerosis on cardiovascular events. Patients with one or more cardiovascular risk factor(s) (obesity, smoking, presence of cardiovascular event history, hypertension, dyslipidemia, diabetes mellitus, chronic kidney disease) and being treated at the Division of Diabetes, Endocrinology and Metabolism, Hyogo Medical College Hospital (Hyogo, Japan) were registered. All agreed to participate in the study by providing written informed consent and the study was approved by the Ethics Committee of Hyogo College of Medicine (approval No. 948).

### 2. Assessment of classical cardiovascular risk factors and medical hypertension treatment

We measured height and body weight, and obtained a medical history. Body mass index (BMI) was calculated as weight in kilograms divided by the square of the height in meters (kg/m^2^). Smoking status was ascertained on the basis of self-reported history of cigarette smoking. We defined cardiovascular events as history of coronary heart disease (myocardial infarction or coronary intervention) or stroke (ischemic or hemorrhagic stroke) diagnosed by computed tomography or magnetic resonance imaging. Dyslipidemia was defined as low density lipoprotein cholesterol level ≥140 mg/dl, high density lipoprotein cholesterol level ≤40 mg/dl, and triglyceride ≥150 mg/dl, or history of treatment for dyslipidemia as previously described [22]. Type 2 diabetes was diagnosed by fasting plasma glucose ≥126 mg/dl, casual plasma glucose ≥200 mg/dl, or 2-hour plasma glucose ≥200 mg/dl during a 75-g oral glucose tolerance test, or previous therapy for diabetes [23]. Serum creatinine (S-Cr) was measured using the enzymatic method. The estimated glomerular filtration rate (eGFR) in each patient was calculated using a new equation for Japanese subjects, as follows: eGFR (ml/min/1.73 m2)  = 194×age−0.287×S-Cr−1.094 (if female, ×0.739) [24]. We defined medical hypertension treatment as taking antihypertensive medications, calcium (Ca) channel blockers, α/β blockers, angiotensin converting enzyme (ACE) inhibitors, angiotensin ΙΙ receptor blockers (ARB), or diuretic agents.

### 3. Sleep apnea-hypopnea index(AHI) measurement

An Apnomonitor (SAS-2100, Teijin, Tokyo, Japan) was used to measure AHI, as previously described [25]. Percutaneous oxygen saturation (SpO2) was recorded using a pulse oximeter. Apnea was defined as complete cessation of air flow lasting ≥10 seconds. Hypopnea was defined as a ≥50% reduction in air flow lasting ≥10 seconds associated with a 4% decrease in oxygen saturation. AHI was defined as the average number of apnea and hypopnea episodes per hour.

### 4. Ambulatory blood pressure monitoring (ABPM)

ABPM was performed with a TM-2431 digital recorder and the obtained data were analyzed with TM-9503 Doctor Pro 3 software (A&D Co.Ltd., Tokyo, Japan) as previously described [26]. Measurements were obtained every 30 minutes over 48 hours (from evening to evening), resulting in daytime and nighttime mean systolic blood pressure (SBP) and diastolic blood pressure (DBP) values, as previously described [18]. Basically, the latter 24 hour-recorded data were used for analyses. The major quality criteria used for an acceptable ABPM recording included the following: (1) minimum of 80% of BP readings expected during the 24-hour period, (2) no more than 2 nonconsecutive hours with <1 valid BP reading, and (3) no behavior seriously affecting BP (afternoon nap, drinking alcohol, etc), as previously described [26]. Wake and sleep times were determined using an Actigraph (Ambulatory Monitoring, Inc., Ardley, New York, USA), as previously described [27]. This device is worn on the wrist of the non-dominant arm and senses motion as acceleration, and then uses standard criteria to identify the onset and offset of sleep periods with a built-in algorithm. Nocturnal SBP fall (%) was calculated as 100× [1-sleep SBP/awake SBP ratio]. We classified the patients by nocturnal SBP fall as follows: extreme-dippers if the fall was ≥20%, dippers if the fall was ≥10% but <20%, non-dippers if it was ≥0% but <10%, and reverse-dippers if it was <0% [18]. The coefficients of variations (9 subjects) of nocturnal SBP fall for repeated 24-hour measurements was 21.2%.

### 4. HRV

HRV analysis is generally used as a noninvasive procedure to measure cardiac modulation by autonomic nervous activity. For that analysis, we used an Active Tracer (AC-301A, Arm Electronics, Tokyo, Japan) to monitor the surface electrocardiogram of the upper limbs for 48 hours via 3 channels, as previously described [28]. The latter 24-hour recorded time series of data was analyzed using MemCalc Chiram 3, version 2.0 (Suwa Trust, Tokyo, Japan). Ectopic beats, noisy data, and artifacts were manually corrected or excluded from the calculations. According to the recommendations for clinical use of HRV [29], the standard deviation of the NN(RR) interval (SDNN), the standard deviation of the average NN(RR) intervals for each 5-minute (SDANN5), and the coefficient of variation R-R interval (CVRR) within the time domain were calculated. SDNN and CVRR were considered to reflect all cyclic components responsible for variability in heart rate [29]. Also, SDANN5 was used as an estimate of change in heart rate due to cycles longer than 5 minutes [29]. Within the frequency domain, values for low frequency (LF; 0.04–0.15 Hz) and high frequency (HF; 0.15–0.4 Hz) power, as well as the ratio of LF to HF (LF/HF) were analyzed by the power spectrum method. Frequency domain analyses contributed to understanding of the autonomic background of the RR interval and power spectrum analysis provided information regarding how power is distributed as a function of frequency. The LF and HF values were considered to reflect sympathovagal balance and parasympathetic nervous activity, respectively [29]. Values for the coefficients of the variations (10 subjects) in repeated 24-hour measurements were as follows: SDNN, 8.2%; SDANN,5 9.6%; CVRR, 6.1%; LF, 28.3%; HF, 15.2%; LF/HF, 0.3%.

### 5. Plasma BDNF

Blood samples were essentially obtained in the morning during recordings with the ABPM and Active Tracer after an overnight fast, and then quickly centrifuged to obtain plasma. Plasma BDNF was measured by an enzyme-linked immunosorbent assay (BDNF Emax ImmunoAssay System kit; Promega Inc., Madison, WI, USA) [30], according to the manufacturer's instructions, with some modifications. Plates (96 wells) were coated with 10 µl of a monoclonal antibody against BDNF in 9.99 ml of a buffer containing 0.025 M sodium bicarbonate and 0.025 M sodium carbonate (pH 9.7) overnight at 4°C. After washing in TBST [20 mM Tris-HCL (pH 7.6), 150 mM NaCl, 0.05% Tween 20], 200 µl of blocking buffer at room temperature was added to the wells for 1 hour. The BDNF standard (1∶2000 dilution) to achieve a concentration of 500 pg/ml, as well as samples (1∶100 dilution) and a blank (no BDNF) were added in triplicate to separate wells. The plates were then incubated for 2 hours at room temperature and washed 5 times with TBST. A polyclonal antibody against BDNF (1∶500 dilution, 20 µl) was added to each well and the plates were incubated for 2 hours at room temperature. After 5 washes in TBST, 50 µl of anti-IgG antibody conjugated with horseradish peroxidase (1∶200 dilution) was added to each well and the plates were incubated for 1 hour at room temperature, then the wells were washed 5 times with TBST. Next, 100 µl of TMB One solution at room temperature was added and incubated for 10 minutes at room temperature, after which the reactions were stopped by adding 100 µl of 1 N hydrochloric acid and absorbance at 450 nm was measured using an automated microplate reader. Standard curves were plotted for each plate. Triplicate measures were averaged and values for total amount of protein in the sample were corrected to derive the pg of BDNF protein/mg of total protein. In this assay system, the intra-assay and inter-assay coefficients of variations (3 subjects) using the same kit lot were 5.7% and 12.4%, respectively.

### 6. Statistical analysis

For the analyses, BDNF and HRV parameters (SDNN, SDANN5, CVRR, LF, HF, LF/HF) were natural logarithm-transformed (ln) to normalize the skewed distribution. To compare variables between groups, a chi-square test (for categorical variables) and non-repeated measures ANOVA F-test (continuous variables with normal distribution) were utilized. A post hoc test was used to compare with dippers with Bonferroni correction. We performed multivariate logistic regression analyses to calculate the odds ratio (OR) and 95% confidence intervals (CI) for BDNF associated with the non- and reverse-dippers as compared to the dippers. All statistical analyses were performed using the Statistical Package for Social Sciences software package (PASW Statistics, version 18.0). All reported p values are 2-tailed and were considered statistically significant at <0.05.

## Results

The distribution of dipping patterns in the patients were as follows: reverse-dippers, 14%; non-dippers, 39%; dippers, 40%; and extreme-dippers, 7% (Table 1). Age was significantly different among the 4 groups, which is in accordance with a previous report [18]. In contrast, other cardiovascular risk factors including gender, smoking, co-morbid conditions (dyslipidemia, diabetes, past cardiovascular diseases) were not statistically significant among the groups, which has been commonly observed in other cohorts [19]–[21]. Sleep SBP and DBP were significantly higher in the non- (P<0.001) and reverse-dippers (P<0.001) as compared to the dippers, while awake DBP was significantly lower in the reverse-dippers (P = 0.009) than the dippers.

**Table 1:** Clinical characteristics of subjects (n = 250).

Variables	Reverse-Dippers	Non-dippers	Dippers	Extreme-Dippers	P
	(n = 36)	(n = 97)	(n = 100)	(n = 17)	
Age	61.7±16.1	59.3±13.4	56.1±14.0	65.8±8.9	0.023
Male sex	18 (50.0%)	54 (55.7%)	58 (58.0%)	8 (47.1%)	0.759
Body mass index (kg/m^2^)	23.7±4.2	24.7±5.4	24.8±4.1	25.2±6.7	0.626
Current smoker (%)	12 (33.3%)	25 (25.8%)	32 (32.7%)	4 (23.5%)	0.645
Past cardiovascular disease (%)	5 (13.9%)	13 (13.4%)	15 (15.0%)	3 (17.6%)	0.967
Dyslipidemia (%)	19 (52.8%)	58 (59.8%)	63 (63.0%)	9 (52.9%)	0.684
Diabetes mellitus (%)	18 (50.0%)	38 (39.2%)	40 (40.0%)	10 (58.8%)	0.337
eGFR (ml/min/1.73 m^2^)	79.8±33.5	79.9±20.3	81.9±25.6	73.9±22.2	0.660
Medical hypertension treatment					
Calcium-channel blocker (%)	14 (38.9%)	47 (48.5%)	41 (41.0%)	6 (35.3%)	0.574
α or β blocker (%)	7 (19.4%)	7 (7.2%)	6 (6.0%)	1 (5.9%)	0.080
ACE inhibitor or ARB (%)	12 (33.3%)	32 (33.0%)	23 (23.2%)	3 (17.6%)	0.294
Diuretic agent (%)	4 (11.1%)	7 (7.2%)	12 (12.0%)	0 (0.0%)	0.351
AHI	9.0±9.7	10.3±12.0	9.4±10.2	10.4±14.4	0.919
ABPM					
SBP (mmHg)					
24-hour	126.7±19.0	125.3±16.4	121.5±11.4	124.9±11.7	0.173
Awake	124.3±19.1	127.8±16.6	127.3±12.2	134.3±11.8	0.170
Sleep	131.5±21.0**	120.5±16.3**	109.3±10.7	101.8±9.9	<0.001
DBP (mmHg)					
24-hour	72.2±9.6	75.9±9.4	73.6±7.7	71.7±5.7	0.057
Awake	71.8±9.8**	77.8±9.7	77.3±8.0	77.5±5.4	0.005
Sleep	73.7±10.1**	71.8±9.2**	65.5±7.6	57.6±7.0**	<0.001
Nocturnal SBP dipping (%)	−5.7±4.4**	5.7±3.0**	14.1±2.6	24.1±4.1**	<0.001

Data are presented as the mean ± standard deviation and number (%) for dichotomous variables. eGFR; estimated glomerular filtration rate, ACE; angiotensin converting enzyme, ARB: angiotensin receptor blocker, AHI; apnea hypopnea index, ABPM; ambulatory blood pressure monitoring, SBP; systolic blood pressure, DBP; diastolic blood pressure. Overall P values represent the 4-group comparison of means (ANOVA F-test) or percentage (chi-square test).

*P<0.05,

**P<0.01 vs. dippers. (post hoc test, Bonferroni correction).

**Figure 1:**
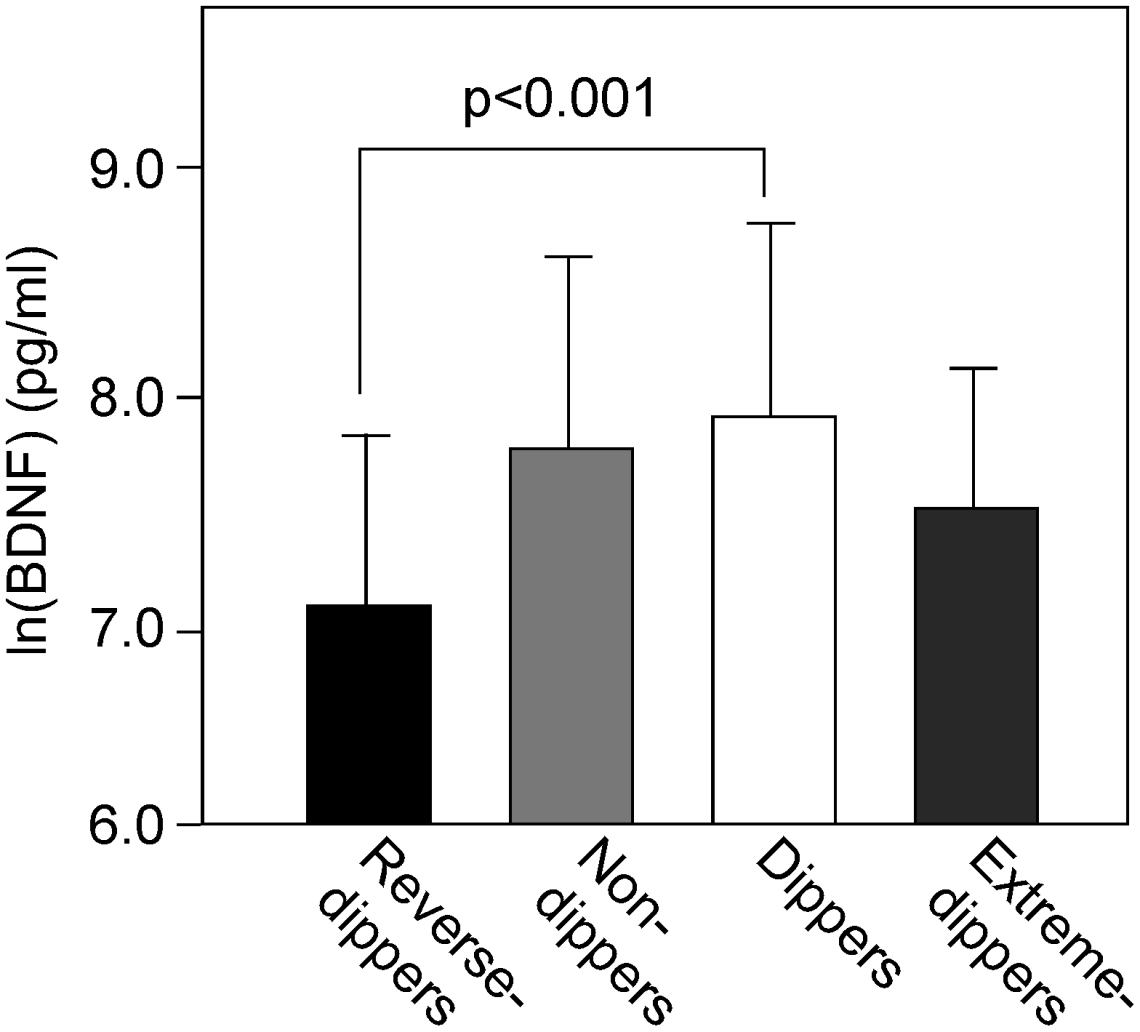
Plasma BDNF in patients divided into extreme-dippers, dippers, non-dippers, and reverse-dippers based on changes in nocturnal blood pressure. BDNF was natural logarithm-transformed (ln) to achieve a normal distribution. Each column represents the mean ± standard deviation. The overall P value for the 4-group comparison of means was calculated using an ANOVA F-test, while a post hoc test with Bonferroni correction was used to compare with dippers. There were significant differences in plasma BDNF among the 4-groups (P<0.001), and its level in the reverse dippers was significantly lower than in the dippers.

Plasma ln(BDNF) levels in the 4 groups are shown in Figure 1 (reverse-dippers, 7.18±0.69, p<0.001; non-dippers, 7.71±0.86 pg/ml; dippers, 7.86±0.86 pg/ml;- extreme-dippers, 7.45±0.69 pg/ml; ANOVA). Plasma BDNF was significantly lower in the reverse-dippers than the dippers (P<0.001). To determine whether plasma BDNF was independently associated with the reverse-dippers, multiple logistic regression analyses were performed using age, gender, classical cardiovascular risk factors (body mass index, current smoking, cardiovascular disease history, dyslipidemia, diabetes mellitus, eGFR), medical hypertension treatment (calcium-channel blocker, α or β blocker, ACE inhibitor or ARB, diuretic agent), AHI and BDNF as covariates (Table 2). Although the level of plasma BDNF was not associated with the non-dippers, it was the sole independent factor and significantly associated with the reverse-dippers when compared with the dippers (as a control). Plasma BDNF level tended to correlate with nocturnal BP fall (r = 0.114, P = 0.072), with the relationship becoming significant when extreme-dippers were excluded from analysis (r = 0.193, P = 0.003). Plasma BDNF was not significantly associated with the awake (r = 0.028, P = 0.667) or sleep (r = −0.091, P = 0.168) SBP values. In the present cohort, plasma BDNF level was not significantly correlated with age, gender, BMI, smoking status, history of cardiovascular diseases, presence of dyslipidemia, hypertension or diabetes (data not shown).

**Table 2:** Plasma BDNF independently associated with reverse-dipper pattern of nocturnal blood pressure change.

	Reverse-dippers		Non-dippers	
Variables	OR (95% CI)	P	OR (95% CI)	P
Age	1.001 (0.957–1.047)	0.968	1.007 (0.977–1.038)	0.642
Male sex (female = 0, male = 1)	0.392 (0.128–1.196)	0.100	0.949 (0.501–1.798)	0.873
Classical cardiovascular risk factors				
Body mass index	0.929 (0.814–1.060)	0.273	0.972 (0.892–1.059)	0.518
Current smoker	1.163 (0.417–3.238)	0.773	0.758 (0.383–1.501)	0.426
(absence = 0, presence = 1)				
Cardiovascular disease history	0.804 (0.204–3.167)	0.756	0.912 (0.368–2.259)	0.842
(absence = 0, presence = 1)				
Dyslipidemia	0.497 (0.178–1.382)	0.180	0.889 (0.463–1.704)	0.722
(absence = 0, presence = 1)				
Diabetes mellitus	2.477 (0.850–7.214)	0.096	0.899 (0.455–1.775)	0.758
(absence = 0, presence = 1)				
eGFR	1.003 (0.985–1.021)	0.767	0.997 (0.983–1.011)	0.671
Medical hypertension treatment				
Calcium-channel blocker	1.267 (0.462–3.479)	0.646	1.273 (0.666–2.434)	0.466
(absence = 0, presence = 1)				
α or β blocker	1.903 (0.308–11.744)	0.489	1.317 (0.316–5.483)	0.169
(absence = 0, presence = 1)				
ACE inhibitor or ARB	1.399 (0.403–4.855)	0.597	1.727 (0.793–3.761)	0.705
(absence = 0, presence = 1)				
Diuretic agent	0.734 (0.140–3.851)	0.714	0.436 (0.141–1.342)	0.148
(absence = 0, presence = 1)				
AHI	1.014 (0.959–1.071)	0.630	1.015 (0.982–1.048)	0.382
ln(BDNF)	0.417 (0.228–0.762)	0.004	0.954 (0.659–1.381)	0.801

Multiple logistic regression analyses were performed. The covariates included age, male sex, classical cardiovascular risk factors (body mass index, current smoking, cardiovascular disease history, dyslipidemia, diabetes mellitus, eGFR), medical hypertension treatment (calcium-channel blocker, α or β blocker, ACE inhibitor or ARB, diuretic agent), AHI and BDNF. BDNF was natural logarithm-transformed (ln) to achieve a normal distribution. OR; odds ratio, CI; confidence interval, eGFR; estimated glomerular filtration rate, ACE: angiotensin converting enzyme, ARB; angiotensin receptor blocker, AHI; apnea hypopnea index, BDNF; brain-derived neurotrophic factor.

To examine the potential involvement of autonomic function in the relationship between BDNF and reverse-dippers, we next examined the associations between BDNF and HRV parameters (Table 3). Simple regression analyses showed that plasma BDNF levels were significantly associated with all of the time-domain measurements of HRV (SDNN, SDANN5, CVRR), and the frequency-domain measurements LF and HF. Moreover, all time-domain HRV parameters and LF were significantly (p<0.05) correlated with nocturnal SBP fall in simple regression analysis findings (SDNN: r = 0.140,SDANN5: r = 0.138, CVRR: r = 0.163, LF: r = 0.201). Furthermore, the relationships of SDNN (β = 0.143), CVRR (β = 0.166), and LF (β = 0.201) with nocturnal BP fall remained significant even after adjusted for covariates including age, gender, BMI, smoking, history of cardiovascular diseases, dyslipidemia, diabetes mellitus, eGFR, hypertension treatments, and AHI. Finally, we examined the impact of inclusion of HRV parameters in the reverse-dippers using multiple logistic regression analyses. The relationship between plasma BDNF and the reverse-dippers was still significant or borderline significant even with ln(SDNN), ln(SDANN5), ln(CVRR), or ln(LF) included as a covariate (Table 4). In this model, ln(LF) was significantly associated with the reverse-dippers and independent of plasma BDNF level.

**Table 3:** Association of plasma BDNF with HRV parameters.

	r	P
Time-domain measurements of HRV		
ln(SDNN)	0.180	0.006
ln(SDANN5)	0.162	0.010
ln(CVRR)	0.194	0.003
Frequency-domain measurements of HRV		
ln(LF)	0.275	<0.001
ln(HF)	0.177	0.007
ln(LF/HF)	0.102	0.123

Pearson's correlation coefficients between the HRV parameters and BDNF were calculated. All parameters of HRV and BDNF were natural logarithm-transformed (ln) to achieve a normal distribution. HRV; heart rate variability, SDNN; standard deviation of the NN(RR) interval, SDANN5; standard deviation of average of NN intervals for each 5 minute period, CVRR; coefficient of variation R-R interval, LF; low frequency power, HF; high frequency power, LF/HF; the ratio of LF to HF, BDNF; brain-derived neurotrophic factor.

**Table 4:** Plasma BDNF and LF independently associated with reverse-dipper pattern of nocturnal blood pressure change.

Variables	OR (95% CI)	P
Model 1		
ln(BDNF)	0.417 (0.228–0.762)	0.004
Model 2		
ln(SDNN)	0.397 (0.070–2.256)	0.297
Model 3		
ln(SDNN)	0.444 (0.071–2.767)	0.384
ln(BDNF)	0.485 (0.259–0.911)	0.024
Model 4		
ln(SDANN5)	0.517 (0.121–2.218)	0.375
Model 5		
ln(SDANN5)	0.587 (0.124–2.789)	0.503
ln(BDNF)	0.421 (0.230–0.772)	0.005
Model 6		
ln(CVRR)	0.286 (0.041–2.015)	0.209
Model 7		
ln(CVRR)	0.339 (0.046–2.508)	0.290
ln(BDNF)	0.489 (0.260–0.920)	0.026
Model 8		
ln(LF)	0.371 (0.177–0.781)	0.009
Model 9		
ln(LF)	0.422 (0.196–0.910)	0.028
ln(BDNF)	0.555 (0.291–1.059)	0.074

Multiple logistic regression analyses were performed. The covariates included age, male sex, classical cardiovascular risk factors (body mass index, current smoking, cardiovascular disease history, dyslipidemia, diabetes mellitus, eGFR), medical hypertension treatment (calcium-channel blocker, α or β blocker, ACE inhibitor or ARB, diuretic agent) and AHI. Each model includes a HRV parameter with or without BDNF in addition to the above covariates. Model 1: + ln(BDNF), Model 2: + ln(SDNN), Model 3: + ln(SDNN) and ln(BDNF), Model 4: + ln(SDANN5), Model 5: + ln(SDANN5) and ln(BDNF), Model 6: + ln(CVRR), Model 7: +ln(CVRR) and ln(BDNF), Model 8: +ln(LF), and Model 9: + ln(LF) and ln(BDNF). The HRV parameters and BDNF were natural logarithm-transformed (ln) to achieve a normal distribution. OR; odds ratio, CI; confidence interval, eGFR; estimated glomerular filtration rate, ACE; angiotensin converting enzyme, ARB; angiotensin receptor blocker, AHI; apnea hypopnea index, SDNN; standard deviation of NN(RR) interval, SDANN5; standard deviation of average of NN intervals for each 5 minute period, CVRR; coefficient of variation R-R interval, LF; low frequency power, BDNF; brain-derived neurotrophic factor.

## Discussion

This study is the first to show a relationship between a reverse-dipping pattern of nocturnal blood pressure and plasma BDNF level, while cardiac autonomic functions may also be involved in that relationship.

Previous studies have suggested that nocturnal hypertension is caused by several different mechanisms, such as abnormal renal sodium handling, nocturnal autonomic imbalance, systemic and vascular inflammation, and endothelial dysfunction, among which autonomic nervous dysfunction might be a leading pathogenetic factor [31]. In this study, we focused on the relationships among plasma BDNF, cardiac autonomic function, and dipping patterns of nocturnal blood pressure in a cohort composed of 250 patients with cardiovascular risk factor(s).

In our study, plasma BDNF level was the highest in the dippers, lowest in the reverse-dippers, and intermediate in the non-dippers, with those in the reverse-dippers significantly lower as compared to the dippers. Thus, higher plasma BDNF indicated a greater fall in nocturnal BP in those 3 groups. Indeed, plasma BDNF level was shown to be significantly correlated with nocturnal BP fall in those patients. On the other hand, plasma BDNF level tended to be lower in the extreme dippers, who had the largest nocturnal BP falls, as compared to the dippers. Although it is not clear at present why the extreme dippers did not have a trend for an association between plasma BDNF and nocturnal BP fall, Grassi et al. [32] suggested that contributions of the autonomic nervous system and baroreflex changes in extreme dippers may be distinct from reverse dippers with nocturnal hypertension. Previously, Golden E et al. showed that plasma BDNF level was positively correlated with DBP in 496 healthy middle-age and elderly subjects [16]. In contrast, we did not find a significant relationship of plasma BDNF with ambulatory awake or sleep BP levels. This lack of correlation might have been because of differences in BP measurements, casual or ABPM, or differences in the subjects, as the present enrolled patients had one or more cardiovascular risk factors including hypertension, and some were receiving treatment with medication. The present findings suggest that nocturnal change in blood pressure, rather than blood pressure itself, is more associated with plasma BDNF, while other clinical parameters including cardiovascular risk factors barely correlate with plasma BDNF.

At present, it is not clear how plasma BDNF level is correlated with nocturnal BP changes in humans, though it may reflect the content of BDNF in the central or peripheral nervous system. In a study of rodents, the cortical level of BDNF was correlated with circulating BDNF concentration [33], while another study showed that BDNF concentration in plasma was unrelated to levels found in the cortex and hippocampus [34]. In cultured rodent myocytes, BDNF at a concentration of 100 ng/ml readily exerted its action [35], suggesting the alternative possibility of direct action of circulating BDNF. Another intriguing possibility is that plasma BDNF level oscillates overnight and could correlate with nocturnal BP changes. However, no known studies have reported normal circadian control of plasma BDNF. Our preliminary results (n = 4) showed that plasma BDNF level at 23:00, 1:00, 3:00, 5:00 and 8:00 was 2260.2±828.4, 1913.2±983.3, 2907.4±1233.6, 2889.5±1438.5, and 2638±611.3 pg/ml, respectively, not indicated a dramatic oscillation. Thus, it may be feasible to speculate that plasma BDNF sets a threshold for sympathetic neurotransmitter release [35], in contrast to direct bursts to regulate autonomic tonus.

Based on animal experiments, BDNF appears to play fundamental roles in regulation of the autonomic nervous system. In the present study, we showed for the first time that plasma BDNF in humans is significantly associated with several parameters of HRV representing cardiac autonomic functions. In rodents, BDNF and its receptor TrkB were found to be highly expressed in the nucleus tractus solitarius and microinjection of BDNF into the nucleus increased nerve activity, while microinjection of K252a (a tyrosine kinase inhibitor) as well as immuno-neutralization of endogenous BDNF with anti-BDNF antibody decreased nerve activity. BDNF was also shown to be involved in development and survival of the arterial baroreceptor system [36]. In a rat model, BDNF was identified as a candidate molecule for the mediator of activity-dependent changes at baroafferent synapses during hypertension [11], while it has also been shown to quickly alter the neurotransmitter release properties of sympathetic neuron-myocyte connections in rodent cell cultures, leading to a rapid shift from excitatory to inhibitory cholinergic transmission in response to neuronal stimulation [35].

Our findings regarding the relationship between plasma BDNF and HRV parameters suggest evidence for the role of BDNF in regulation of autonomic nervous system, and also suggest that the autonomic nervous system mediates the association between BDNF and the reverse dipper pattern. Sympathetic activation appears to represent a mechanism potentially responsible for day-night BP changes [19], [21]. In our cohort, many of the HRV parameters, including SDNN, SDANN5, CVRR, and LF, were significantly associated with nocturnal SBP fall. A previous limited study also found a relationship between HRV parameters and the nocturnal BP patterns. Takagi et al. [20] investigated the relationship among 24-hour, nighttime, and daytime LF and HF components using spectral analyses in patients with hypertension, and reported that 24-hour and daytime LF/HF and LF were significantly lower in reverse-dippers as compared to dippers, even though the time-domain parameters of HRV were not included in their analyses. As shown in Table 2, plasma BDNF was the sole significant factor associated with the reverse-dippers in our multiple logistic regression analysis that included age, gender, classical cardiovascular risk factors, medical hypertension treatments, and AHI as covariates. Among the covariates, age was one of the strongest determinants for decreases in HRV parameters, as previously reported [37]. Diabetes [38], chronic kidney disease [39], and sleep disorders [40] have also been shown to be related to autonomic dysfunction. Importantly, the association between plasma BDNF and the reverse-dippers was rather weakened, yet remained significant or borderline significant even after further adjustment for all HRV parameters (SDNN, SDANN5, CVRR, LF) which were significantly associated with plasma BDNF in simple regression analyses. Thus, unveiled mechanisms may still exist for the relationship between plasma BDNF and reverse-dippers, except for the role of autonomic nervous dysfunction in sleep BP fall.

The present study has several limitations. First, because of ethical considerations, our subjects were analyzed for patterns of nocturnal blood pressure changes without discontinuation of antihypertensive medication, which is generally performed for ABPM measurements in a healthy population. Second, day-to-day BP measurements might be very much variables, since CV for measuring nocturnal BP changes for 2 consecutive days was relatively high (21.2%), while change in the patterns of dipping of nocturnal BP may also be variable. Thus in this study, to minimize the influence of day-to-day variations, ABPM and HRV were simultaneously measured, with plasma BDNF measured during the corresponding morning. Third, diabetic neuropathy was not included as a covariate in this study, because quantitative nerve conduction velocity were not measured in all of the diabetic subjects. Diabetes neuropathy has been shown to be associated with nocturnal hypertension [41]. Fourth, because of the study design, the potential nocturnal oscillation of plasma BDNF was not considered in this cohort study. Finally, frequency-domain measurements of HRV with spectral analyses of non-stationary ECG recording may be affected by noise sources [29], and day-to-day variations can be very large. However, both time- and frequency-domain measurements of HRV showed similar results in our cohort, suggesting a contribution of cardiac autonomic function.

Regardless of these potential limitations, our findings are the first to show a mutual relationship among BDNF, the autonomic nervous system, and reverse-dippers of nocturnal hypertension, and provide an important first step to answer pressing pathophysiological questions in regard to hypertension.
